# Efficacy and safety of traditional Chinese medicine Elian Granule for chronic atrophic gastritis: a multi-center, randomized, double-blind, placebo-controlled study

**DOI:** 10.3389/fphar.2025.1545313

**Published:** 2025-04-28

**Authors:** Qingling Jia, Yuebo Jia, Yongqi Chen, Likun Guo, Chenheng Wu, Jun Cong, Zhijian Gu, Xuejun Li, Shengquan Fang, Zhuhua Liu, Kailin Jiang, Xuejiao Liu, Gan Cai, Xudong Tang, Jianghong Ling

**Affiliations:** ^1^ Department of Gastroenterology, Shuguang Hospital, Shanghai University of Traditional Chinese Medicine, Shanghai, China; ^2^ Department of Pathology, Shuguang Hospital, Shanghai University of Traditional Chinese Medicine, Shanghai, China; ^3^ Endoscopy Center, Shuguang Hospital, Shanghai University of Traditional Chinese Medicine, Shanghai, China; ^4^ Department of Spleen and Stomach Diseases, The Second Affiliated Hospital of Anhui University of Traditional Chinese Medicine, Hefei, Anhui, China; ^5^ Department of Gastroenterology, Yueyang Hospital of Integrated Traditional Chinese and Western Medicine, Shanghai University of Traditional Chinese Medicine, Shanghai, China; ^6^ Department of Spleen and Stomach Diseases, Affiliated Hospital of Shanxi University of Chinese Medicine, Taiyuan, Shanxi, China; ^7^ Institute of Spleen and Stomach Diseases, Xiyuan Hospital of China Academy of Chinese Medical Sciences, Beijing, China

**Keywords:** Elian Granule, traditional Chinese medicine, chronic atrophic gastritis, clinical efficacy, clinical safety

## Abstract

**Objective:**

This multi-center, randomized, double-blind, placebo-controlled study aimed to evaluate the clinical efficacy and safety of Elian Granule in treating chronic atrophic gastritis (CAG).

**Methods:**

Over a 24-week period, 240 CAG patients were randomized to receive either Elian Granule or placebo. Primary outcomes included histological improvement of gastric mucosa via biopsy, while secondary outcomes assessed dyspepsia symptom scores and quality of life (QOL) scores. Safety was monitored through physical examinations, laboratory tests (blood and urine tests, liver function, and renal function), and electrocardiograms (ECGs).

**Results:**

The Elian Granule group exhibited significantly higher improvement rates in gastric mucosal atrophy (76.29% vs. 48.96%, *P* < 0.001) and intestinal metaplasia (62.89% vs. 34.38%, *P* < 0.001) compared to the placebo group. Total dyspepsia scores improved at 4, 12, and 24 weeks (*P* < 0.001), the individual symptom scores showed significant improvement in epigastric pain, epigastric distension, epigastric discomfort, early satiety, heartburn, belching and acid reflux at both the 4-week and 12-week timepoints (*P* < 0.05, *P* < 0.01, *P* < 0.001), of these, epigastric pain, epigastric distension, early satiety, belching and acid reflux maintained their statistically significant improvement through the 24-week evaluation period (*P* < 0.05, *P* < 0.01, *P* < 0.001). The total effective rate for symptom relief was 85.57% in the Elian group versus 47.92% in the placebo group (*P* < 0.001). QOL scores for physical health (GH, PF, BP, and PCS total score) and mental health (VT, SF, MH, and MCS total score) also improved significantly (*P* < 0.05, *P* < 0.01). No adverse impact was observed.

**Conclusion:**

Elian Granule significantly improves gastric mucosal atrophy and intestinalization, alleviates dyspeptic symptoms, and enhances QOL in CAG patients, demonstrating a favorable safety profile.

**Clinical Trial Registration:**

http://itmctr.ccebtcm.org.cn/zh-CN/Home/ProjectView?pid=7a00ecee-da6a-4939-bae1-e6a58cd97cdb, identifier ChiMCTR2000003929, 2020-9-13.

## 1 Introduction

Chronic atrophic gastritis (CAG) is a chronic inflammatory disease characterized by progressive reduction or disappearance of gastric mucosal glands and mucosal layer thinning, classified as a precancerous condition. According to 2022 global cancer statistics, China ranks first worldwide in overall cancer incidence and mortality, with gastric cancer (GC) exhibiting particularly alarming rates - an incidence of 39.7 per million and mortality of 28.9 per million, positioning it as the third most prevalent cancer globally. Uncontrolled inflammatory responses induce persistent damage to normal gastric mucosa, triggering precancerous lesions including atrophy, intestinal metaplasia, and dysplasia - key drivers of malignant progression to GC (Xu et al., 2024; Nathan and Ding, 2010). Notably, CAG patients with concurrent intestinal metaplasia and dysplasia demonstrate a 6% GC incidence, underscoring the critical importance of CAG management in reducing GC morbidity and mortality (de Vries et al., 2008; Shah et al., 2021). Dyspepsia, manifesting as bloating, nausea, belching, and postprandial fullness, represents a hallmark symptom complex in CAG patients.

Current CAG management strategies primarily involve *Helicobacter pylori* (Hp) eradication, gastric acid suppression, anti-bile reflux therapy, antioxidant administration, and mucosal protection. While demonstrating short-term efficacy, these approaches fail to reliably halt atrophy progression (Dinis-Ribeiro et al., 2012). Studies have shown that Chinese medicine has a good clinical effect on CAG. A meta-analysis of 12 studies involving 1,678 patients with Moluodan in the treatment of CAG found that Moluodan could effectively improve the pathologic changes of gastric mucous tissue and clinical symptoms. Another study of 26 randomized controlled trials of Banxia Xiexin Decoction for CAG including 1,985 patients showed that Banxia Xiexin Decoction was also effective in treating CAG (Cao et al., 2020). However, based on the “Consensus Opinion on Chronic Gastritis in China,” Traditional Chinese Medicine (TCM) can be used for CAG, but this is only a conditional recommendation because of the low quality of evidence (Fang et al., 2017). The main problem is that high-quality clinical studies are limited and evidence of multi-center, controlled, and large-sample trials is lacking, therefore, a large sample, multi-center control of TCM clinical study is the key point of relevant research work.

Professor Gan Cai, a nationally renowned TCM expert specializing in digestive disorders, proposes that CAG pathogenesis follows a “deficiency-excess” pattern: spleen-stomach deficiency as the root cause, with phlegm-dampness, heat-toxin, and qi-blood stasis as secondary manifestations. Based on this theory, Professor Cai developed Elian Granule, a formulation comprising 12 Traditional Chinese herbs verified via MPNS (http://mpns.kew.org): *Curcuma phaeocaulis Valeton* (Ezhu), *Coptis chinensis Franch* (Huanglian), *Salvia miltiorrhiza Bge* (Danshen), *Scleromitrion diffusum (Willd.) R.J.Wang* (Baihuasheshecao), *Angelica sinensis (Oliv.) Diels* (Danggui), *Codonopsis pilosula (Franch.) Nannf* (Dangshen), *Glycyrrhiza uralensis Fisch. ex DC* (Gancao), *Atractylodes macrocephala Koidz* (Baizhu), *Pinellia ternata (Thunb.) Makino* (Banxia), *Poria cocos (Schw.) Wolf* (Fuling), *Taraxacum mongolicum Hand. -Mazz* (Pugongying), and *Citrus reticulata Blanco* (Chenpi). This formula synergistically strengthens spleen function, activates blood circulation, clears heat-toxin, and eliminates dampness-phlegm. With over three decades of clinical application, Elian Granule has demonstrated remarkable efficacy: a 6-month trial showed superior improvement rates for intestinal metaplasia (77.27% vs. 45.83%) and atrophy (73.3% vs. 46.7%) compared to Wei Fu Chun (Cong et al., 2015). Subsequent research in gastric precancerous lesions (PLGC, n = 60) revealed significantly better symptom scores, mucosal pathology improvements, and enhanced immune parameters (CD3^+^T cells, CD4^+^T cells, CD4^+^/CD8^+^ ratio) versus Rebapatide (Yang et al., 2021). In addition, our previous experimental studies verified that Elian Granule can mitigate PLGC through NF-κB signaling pathway or JAK2/STAT3 signaling pathway (Yi et al., 2023; Jia et al., 2025). Besides, we have identified the specific targets of the active ingredient of Elian Granule that intervene in PLGC using network pharmacology (Yi et al., 2022).

Building on this foundation, our multicenter randomized double-blind placebo-controlled trial evaluates Elian Granule’s therapeutic effects on CAG through standardized histopathological assessment, dyspepsia symptom scoring, and quality-of-life metrics, while monitoring safety parameters. This study not only perpetuates Professor Cai’s academic legacy but also provides robust evidence for TCM’s role in CAG management.

## 2 Materials and methods

### 2.1 Study design

This multicenter, randomized, double-blind, placebo-controlled trial was conducted across 14 hospitals in China, including Shuguang Hospital Affiliated to Shanghai University of TCM, Yueyang Hospital of Integrated Traditional Chinese and Western Medicine Affiliated to Shanghai University of TCM, The First Affiliated Hospital of Guangxi University of Chinese Medicine, The Affiliated Hospital to Changchun University of Chinese Medicine, The Second Affiliated Hospital of Anhui University of Chinese Medicine, Affiliated Hospital of Shanxi University of TCM, Hangzhou Hospital of TCM, Shanghai Pudong Guangming Hospital of TCM, Shanghai Guanghua Hospital of Integrated Traditional Chinese and Western Medicine, The Second Affiliated Hospital of Anhui Medical University, Shanghai Jiading District TCM Hospital, Traditional Chinese Hospital of Lu’An, Qinghai Red Cross Hospital, and Hubei People’s Hospital of Ma Cheng City Affiliated Hospital of Hubei University of Science and Technology. However, Qinghai Red Cross Hospital, and Hubei People’s Hospital of Ma Cheng City Affiliated Hospital of Hubei University of Science and Technology withdrew due to the pandemic’s impact. Therefore, patient enrollment was conducted across the remaining 12 centers using a competitive enrollment approach between October 2020 to October 2022. The clinical trial protocol was published in 2022 (Gu et al., 2022).

### 2.2 Inclusion criteria

Patients were diagnosed with CAG referring to the “Consensus Opinion on Chronic Gastritis in China (2017, Shanghai)” (Fang et al., 2017) formulated by the Branch of Gastroenterology of the Chinese Medical Association. The criteria for participant inclusion in this study were as follows: (1) individuals identified as male or female, aged between 18 and 70 years, who met the diagnostic criteria for CAG; (2) negative for Hp (at least one C13 or C14 breath test was required); (3) operative link for gastritis assessment/operative link for gastric intestinal metaplasia assessment (OLGA/OLGIM) stages on II-III; (4) willing to participate in this clinical trial with informed consent.

### 2.3 Exclusion criteria

(1) Combined with any significant digestive or medical conditions (peptic ulcer, dysplasia of gastric mucosa or suspected cancer by pathological diagnosis); (2) A history of severe digestive disease or abdominal surgery; (3) Pregnant and lactating women; (4) Patients with severe primary diseases such as heart, lung, hematopoietic system, and malignant tumors, severe diabetes mellitus, chronic liver and renal dysfunction (ALT > 1.5 times the upper limit of normal, and Scr > 1.5 times the upper limit of normal); (5) Platelets lower than normal values; (6) Disabilities defined by law (blind, deaf, mute, intellectual disability, and physically disabled); (7) Long-term using of nonsteroidal anti-inflammatory (NSAID) drugs; a history of alcoholism; (8) Having a history of allergy to multiple drugs; (9) Participating in other clinical trials within the past 4 weeks; use of other traditional Chinese medicines (e.g., Weifuchun, Moluodan), or folic acid and other drugs within the past 2 weeks; (10) Patients deemed by the researcher as likely to be lost to follow-up or otherwise unsuitable for inclusion.

### 2.4 Suspension criteria

The criteria for suspension were as follows: (1) if serious safety problems occur during the experiment, and the researcher determines that the safety of the subjects may be compromised, the trial should be suspended promptly; (2) suspension requested by the sponsor for reasons such as funding reasons or management reasons, etc.

### 2.5 Screening and group assignment

Participants meeting the criteria were randomly assigned in a 1:1 ratio to either the Elian Granule group or the placebo group for a 24-week period. Randomization was performed using block randomization method via SAS statistical software. Before the baseline assessment, an independent study coordinator randomly assigned participants to interventions. Group assignments were concealed in sequentially numbered, opaque sealed envelopes. Interviewers distributed drugs to participants. Elian Granule and the placebo, which contained 5% Elian Granule, were manufactured and packaged by Jiangyin Tianjiang Pharmaceutical Co., Ltd. in China. Both products were indistinguishable. Electronic data capture (EDC), and the REDCap system were used for data acquisition, management, and synchronous entry. Before data logging, Researchers underwent EDC training for REDCap system operation, and collected data based on medical records and observation sheets, ensuring accurate, complete, and timely entry into REDCap. Any issue should be promptly reported and addressed. The inspector was responsible for checking that the test was strictly following the protocol. Confirm that all data are entered synchronously, filled in correctly and completely, and consistent with the original data. Data was checked and evaluated until the EDC unblinding of data entry for the last patient. To ensure statistical accuracy, both participants and research staff involved in the evaluation, data entry, and data analysis were blinded, with disclosure only occurring post-data collection or in the event of significant adverse events (AEs) authorized by the steering committee.

### 2.6 Interventions

Elian Granule (Manufacturer: Jiangyin Tianjiang Pharmaceutical Co., Ltd. of China, Batch No.: 2006338) consists of 12 Chinese herbs: 15 g of *Curcuma phaeocaulis Valeton [Zingiberaceae; Curcuma phaeocaulis Valeton Rhizoma]* (Ezhu), 3 g of *Coptis chinensis Franch [Ranunculaceae; Coptis chinensis Franch Rhizoma]* (Huanglian), 10 g of *Salvia miltiorrhiza Bge [Lamiaceae; Salvia miltiorrhiza Bge Radix et Rhizoma]* (Danshen), 30 g of *Scleromitrion diffusum (Willd.) R.J.Wang [Rubiaceae; Scleromitrion diffusum (Willd.) R.J.Wang All]* (Baihuasheshecao), 10 g of *Angelica sinensis (Oliv.) Diels [Apiaceae; Angelica sinensis (Oliv.) Diels Radix]* (Danggui), 10 g of *Codonopsis pilosula (Franch.) Nannf [Campanulaceae; Codonopsis pilosula (Franch.) Nannf Raidx]* (Dangshen), 6 g of *Glycyrrhiza uralensis Fisch. ex DC [Fabaceae; Glycyrrhiza uralensis Fisch. ex DC Radix et Rhizoma]* (Gancao), 10 g of *Atractylodes macrocephala Koidz [Asteraceae; Atractylodes macrocephala Koidz Rhizoma]* (Baizhu), 10 g of *Pinellia ternata (Thunb.) Makino [Araceae; Pinellia ternata (Thunb.) Makino Rhizoma]* (Banxia), 12 g of *Poria cocos (Schw.) Wolf* (Fuling), 30 g of *Taraxacum mongolicum Hand. -Mazz [Asteraceae; Taraxacum mongolicum Hand. -Mazz all]* (Pugongying), and 6 g of *Citrus reticulata Blanco [Rutaceae; Citrus reticulata Blanco peel]* (Chenpi) (Table 1). The granules were packaged into two bags before serving. All botanical origins were verified against the Chinese Pharmacopoeia (2020) or MPNS (http://mpns.kew.org). The chemical ingredients has been identified from Elian Granule by ultra-performance liquid chromatography-tandem mass spectrometry (UPLC-MS) in our previous study (Yi et al., 2023). Placebo granules contained 5% Elian Granule combined with edible lactose, bitterant, starch, and pigment. Participants in each group received maintenance treatment lasting 24w (Elian Granule solution with 24.2 g (12.1 g/bag) of Elian Granule dissolved in 200 mL water twice daily, or matching placebo). Dyspepsia symptom score and physical examination (temperature, pulse, respiration, blood pressure, etc.) were ordered before the treatment and at 4w, 12w, 24w. Gastric mucosal biopsy histological examination, QOL score, and laboratory tests and electrocardiogram (ECG) were ordered before and at the end of the treatment. All experimental drugs were manufactured under good manufacture practices (GMP).

**TABLE 1:** Phytochemical profile of Elian Granule.

Latin name	Chinese name	Medicinal part	Dosage (g)	Granule weight (g)
*Curcuma phaeocaulis Valeton*	Ezhu	Rhizoma	15	1.1
*Coptis chinensis Franch*	Huanglian	Rhizoma	3	0.5
*Salvia miltiorrhiza Bge*	Danshen	Radix et Rhizoma	10	3.2
*Scleromitrion diffusum (Willd.) R.J.Wang*	Baihuasheshecao	All	30	4.3
*Angelica sinensis (Oliv.) Diels*	Danggui	Radix	10	6.5
*Codonopsis pilosula (Franch.) Nannf*	Dangshen	Radix	10	9
*Glycyrrhiza uralensis Fisch. ex DC.*	Gancao	Radix et Rhizoma	6	1.1
*Atractylodes macrocephala Koidz*	Baizhu	Rhizoma	10	6.6
*Pinellia ternata (Thunb.) Makino*	Banxia	Rhizoma	10	4
*Poria cocos (Schw.) Wolf*	Fuling	Sclerotia	12	4
*Taraxacum mongolicum Hand. -Mazz*	Pugongying	All	30	5.9
*Citrus reticulata Blanco*	Chenpi	Peel	6	2.2
Total granule weight (12.1 g/bag)				48.4

### 2.7 Sample size

The study referenced a phase II clinical trial with 100 cases per group. Anticipating a 20% dropout or loss-to-follow-up rate during the trial, the sample size was increased to 120 cases per group, totaling 240 cases across both groups (Xie et al., 2012).

### 2.8 Randomization and blinding

Eligible patients were randomly assigned to two groups (Elian Granule group and placebo group) in a 1:1 ratio. An independent statistician, unrelated to the investigation, used the PROC PLAN statement in SAS software for drug coding. Eligible patients received individually numbered medication packs.

### 2.9 Outcomes and measurements

Primary outcome was histological examination of gastric mucosal biopsy via gastroscopy, assessed before and after 24-week treatment. Atrophy and intestinal metaplasia were classified as improved, stable or progressive based on OLGA/OLGIM. Effectiveness was defined as a decrease in stage; stability as no change; and progression as an increase in stage.

Secondary outcomes included the dyspepsia symptom score, Quality of life (QOL) score. The dyspepsia symptom score was assessed at baseline (T0), 4w (T1), 12w (T2), and 24w (T3). Dyspepsia symptoms (epigastric pain, epigastric distension, epigastric discomfort, early satiety, belching, heartburn, acid reflux, and abdominal discomfort) were scored as “no” 1, “mild” 2, “moderate” 3, “severe” 4, and “extremely severe” 5 (Gu et al., 2022). The effective rate was calculated as (pre-treatment integral - post-treatment integral)/pre-treatment integral × 100%. The curative effect classification was as follows: ≧95% indicates clinically cured; ≧70% indicates significantly effective; ≧30% indicates effective; <30% indicates ineffective. The total effective rate = clinically cured rate + significantly effective rate + effective rate. QOL was performed at T0 and T3. The internationally recognized short form-12 (SF-12) was used, including general health (GH), physical functioning (PF), role-physical (RP), bodily pain (BP), role-emotional (RE), mental health (MH), vitality (VT), and social functioning (SF). Among them, GH, PF, RP, and BP belong to the physical component summary (PCS), and the rest belong to the mental component summary (MCS) (Gu et al., 2022).

### 2.10 Safety

Safety assessments included vital signs (temperature, pulse, respiration, and blood pressure) measured at baseline, 4, 12, and 24 weeks, along with ECGs and laboratory tests (blood cell count, urinalysis, liver function ALT, AST, ALP, γ-GT, TBIL, and renal function Scr, BUN, GFR). These tests were conducted pre- and post-treatment. Researchers evaluated the patients’ condition and drug safety based on clinical symptoms and test results.

### 2.11 Statistical analysis and data management

Data were analyzed using SPSS 27.0 software. Continuous data were presented as mean (SD), and categorical data were presented as proportion and number as appropriate. Comparisons were analyzed with the use of Student’s t tests for continuous data and Chi-square tests for categorical data. The data from two groups at different time points were compared using ANOVA for repeated measurement. All statistical tests were two-tailed with a 5% significance level.

## 3 Results

### 3.1 Participants and baseline characteristics

**FIGURE 1:**
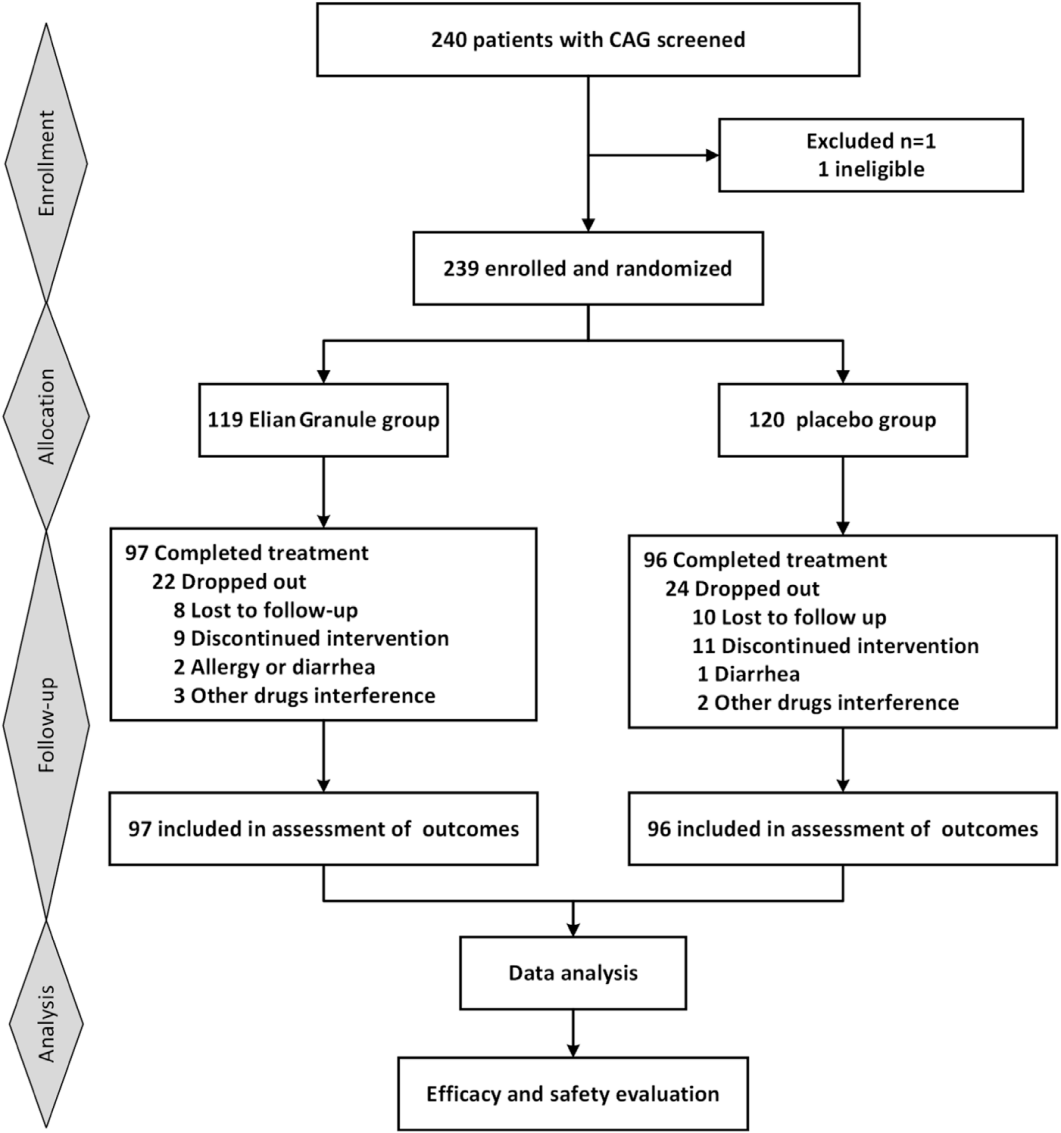
Consort diagram. Consort, consolidated standards of reporting trials.

Initially, 240 participants were planned for enrollment, with 239 eventually included (119 in the Elian Granule group and 120 in the placebo group). Ultimately, 193 participants completed the 24-week intervention (Figure 1). During the trial, 18 participants were lost to follow up, and 28 participants withdrew due to non-trial-related issues, resulting in a 19.25% attrition rate. No significant baseline differences in clinical characteristics were observed between the two groups (Table 2).

**TABLE 2:** Baseline characteristics.

Characteristics	Classification	Placebo (n = 120)	Elian Granule (n = 119)	*P*
Age (mean [SD])		57.35 (9.49)	55.49 (9.82)	0.137
Gender, n (%)				0.897
Female		59 (49.17)	57 (47.90)	
Male		61 (50.83)	62 (52.10)	
Atrophy/intestinal metaplasia, n (%)	Stage II	87 (73.73)	75 (64.64)	0.157
	Stage III	31 (26.27)	41 (35.34)	
Dyspepsia symptom score (mean [SD])		17.34 (5.75)	18.57 (5.90)	0.146
Epigastric pain		2.27 (1.13)	2.46 (1.16)	0.244
Epigastric distension		2.29 (1.12)	2.53 (1.14)	0.152
Epigastric discomfort		2.57 (1.09)	2.63 (1.18)	0.732
Early satiety		1.80 (1.10)	2.05 (1.25)	0.144
Belching		2.23 (1.22)	2.34 (1.16)	0.518
Heartburn		2.01 (1.05)	2.18 (1.16)	0.303
Acid reflux		2.01 (1.04)	2.13 (1.13)	0.431
Abdominal discomfort		2.16 (1.25)	2.25 (1.26)	0.614
PCS score (mean [SD])		2.42 (2.14)	2.85 (2.25)	0.130
GH		−0.30 (0.72)	−0.24 (0.65)	0.465
PF		0.88 (1.42)	1.19 (1.62)	0.117
RP		1.17 (0.78)	1.21 (0.75)	0.724
BP		0.66 (0.72)	0.68 (0.69)	0.806
MCS score (mean [SD])		−17.83 (8.30)	−19.08 (8.44)	0.249
VT		−1.50 (1.20)	−1.68 (1.40)	0.286
SF		−1.68 (2.41)	−2.02 (2.74)	0.307
RE		−6.98 (3.94)	−6.87 (3.88)	0.816
MH		−7.67 (4.95)	−8.51 (5.07)	0.193

### 3.2 Primary outcomes

**FIGURE 2:**
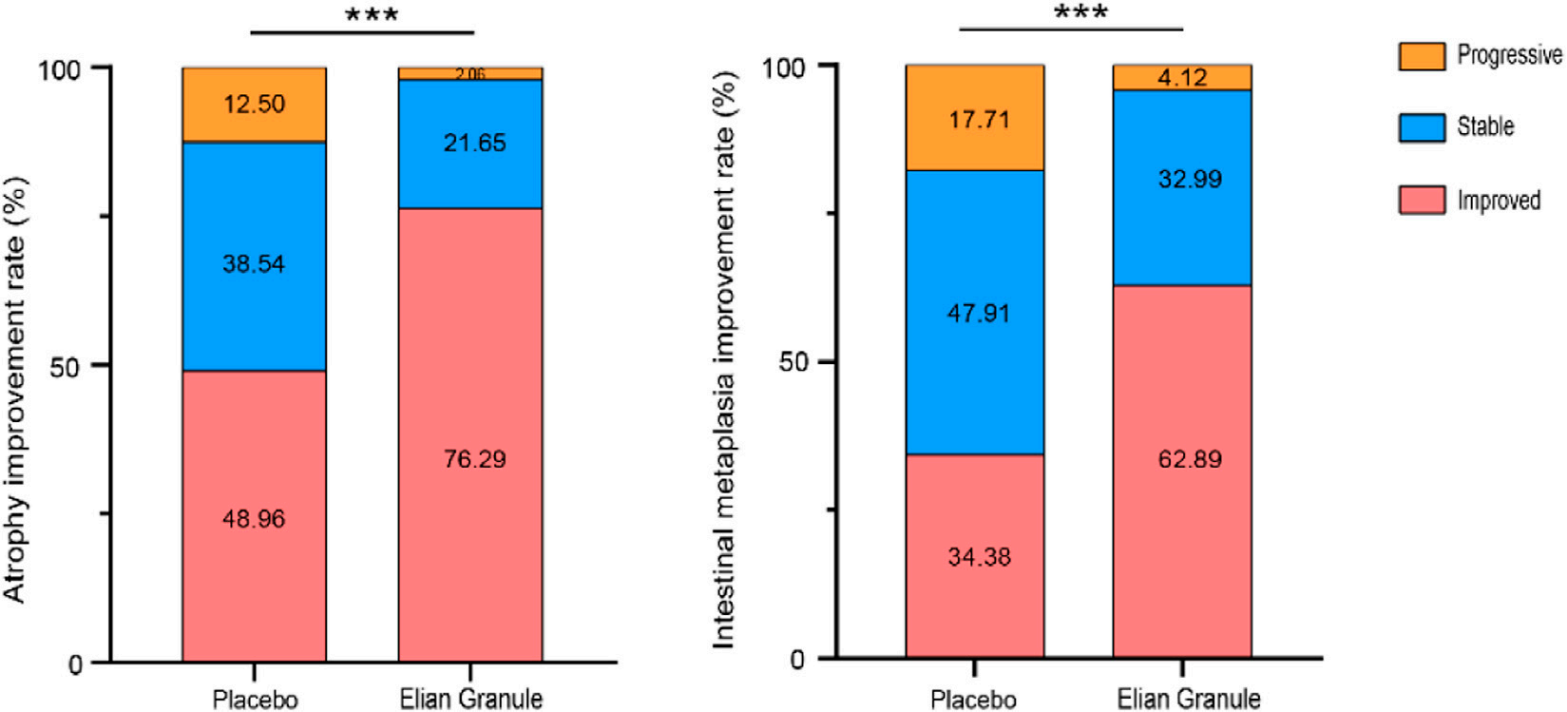
The atrophy improvement rate and intestinal metaplasia improvement rate, compared with the placebo group, ^***^ *P* < 0.001.

**FIGURE 3:**
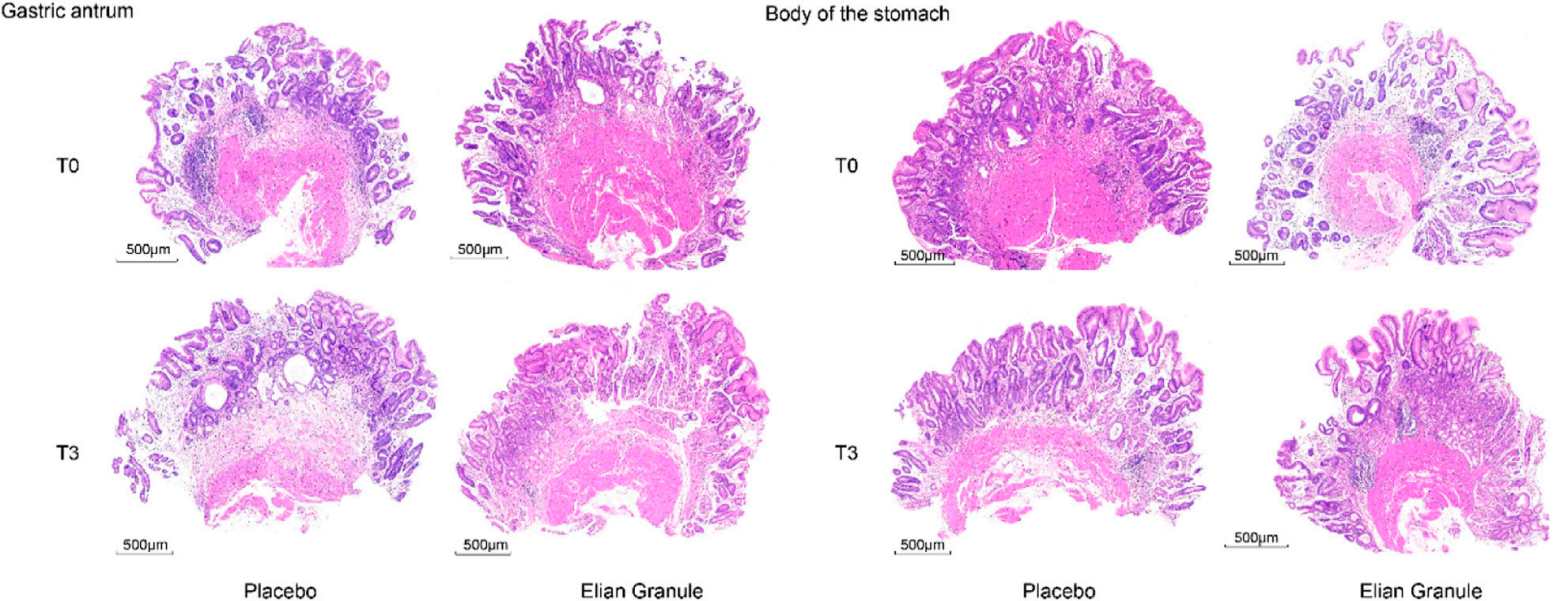
Pathology of the gastric antrum: placebo group: T0---atrophy +++, intestinal metaplasia ++, T3---atrophy +++, intestinal metaplasia ++; Elian Granule group: T0---atrophy +++, intestinal metaplasia +++, T3---atrophy +, intestinal metaplasia +. Pathology of the body of the stomach: placebo group: T0---atrophy +++, intestinal metaplasia +, T3---atrophy ++, intestinal metaplasia ++; Elian Granule group: T0---atrophy ++, intestinal metaplasia +, T3---atrophy +, intestinal metaplasia +. Scare bar: 500 μm.

The atrophy improvement rate was 76.29% (74/97) in the Elian Granule group, compared to 48.96% (47/96) in the placebo group (*P* < 0.001). The intestinal metaplasia improvement rate was 62.89% (61/97) in the Elian Granule group, compared to 34.38% (33/96) in the placebo group (*P* < 0.001), see Figure 2 and Table 3, and pathological selections are detailed in Figure 3.

**TABLE 3:** The atrophy improvement rate and intestinal metaplasia improvement rate.

Outcome	Index	Placebo (n = 96)	Elian Granule (n = 97)	*χ* ^2^	*P*
Atrophy improvement, n (%)	Improved	47 (48.96)	74 (76.29)	17.577	<0.001
	Stable	37 (38.54)	21 (21.65)		
	Progressive	12 (12.50)	2 (2.06)		
Intestinal metaplasia improvement, n (%)	Improved	33 (34.38)	61 (62.89)	18.896	<0.001
	Stable	46 (47.91)	32 (32.99)		
	Progressive	17 (17.71)	4 (4.12)		

### 3.3 Secondary outcomes

**FIGURE 4:**
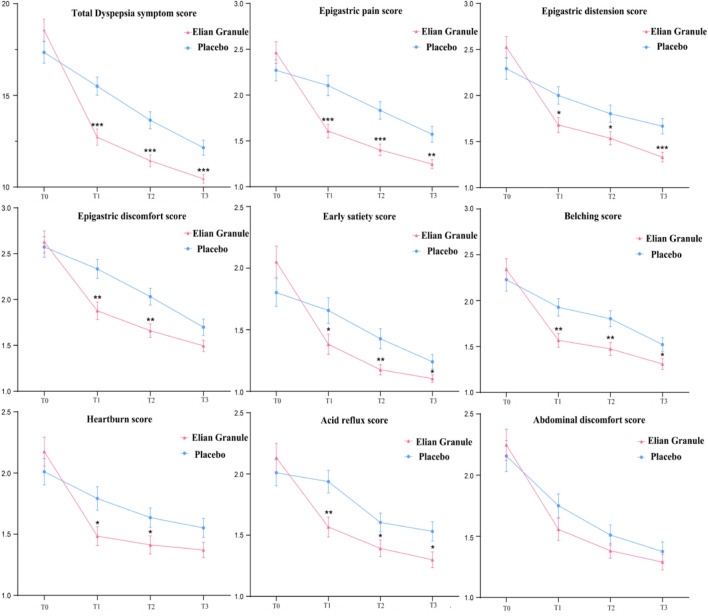
Dyspepsia symptom scores at different time points (^*^ *P* < 0.05; ^**^ *P* < 0.01; ^***^ *P* < 0.001).

Statistical analysis using repeated measures ANOVA revealed significant effects on dyspepsia symptom scores across treatment timepoints (*P* < 0.001), Significant group-by-time interactions (*P* < 0.001) and between-group differences (*P* < 0.01) were observed (Supplementary Material 1). Comparison of the total score of dyspepsia symptoms at different timepoints: there was no significant difference between the two groups before treatment (*P* > 0.05); compared with the placebo group, the total scores of 4w (T1), 12w (T2) and 24w (T3) in the Elian Granule group were significantly reduced (*P*_T1_ < 0.001, *P*_T2_ < 0.001, *P*_T3_ < 0.001). Compared with the placebo group, the subscores of epigastric pain (*P*_T1_ < 0.001, *P*_T2_ < 0.001, *P*_T3_ < 0.01), epigastric distension (*P*_T1_ < 0.05, *P*_T2_ < 0.05, *P*_T3_ < 0.001), early satiety (*P*_T1_ < 0.05, *P*_T2_ < 0.01, *P*_T3_ < 0.05), belching (*P*_T1_ < 0.01, *P*_T2_ < 0.01, *P*_T3_ < 0.05), and acid reflux (*P*_T1_ < 0.01, *P*_T2_ < 0.05, *P*_T3_ < 0.05) in Elian Granule group were significantly decreased at 4w, 12w and 24w treatment. Besides, the scores of epigastric discomfort (*P*_T1_ < 0.01, *P*_T2_ < 0.01), heartburn (*P*_T1_ < 0.05, *P*_T2_ < 0.05) in Elian Granule group were significantly decreased at 4w, and 12w treatment (Figure 4) (Supplementary Material 1).

**FIGURE 5:**
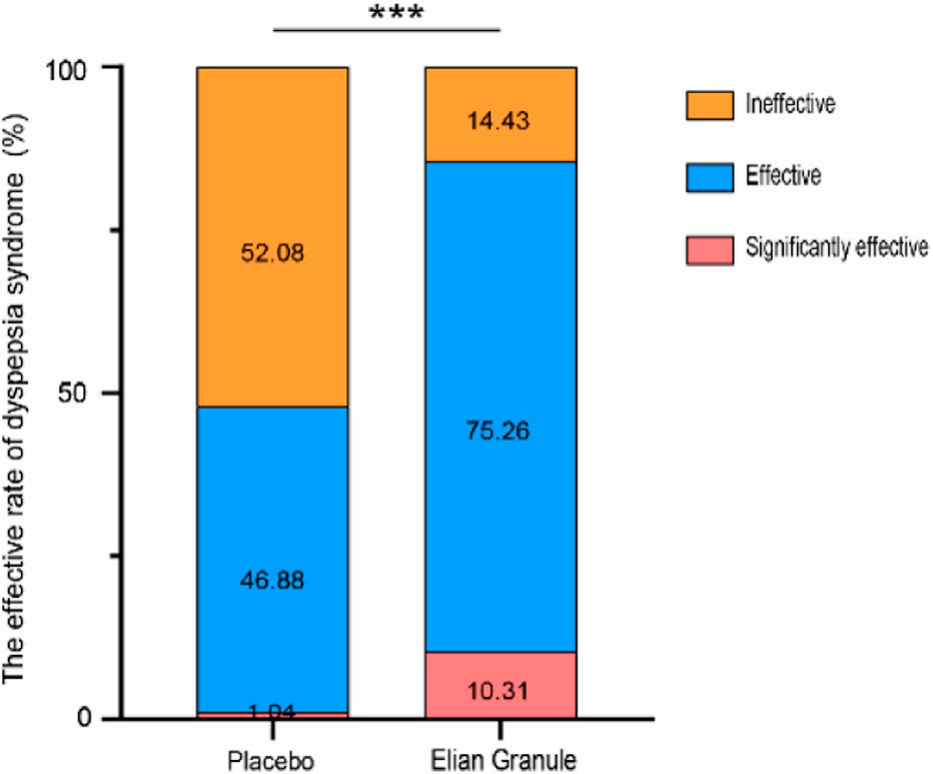
The effective rate of dyspepsia syndrome, compared with the placebo group, ^***^ *P* < 0.001.

Comparison of efficacy of dyspepsia symptoms in the Elian Granule group showed a significant efficiency of 10.31%, an effective rate of 75.26%, and a total effective rate of 85.57%, while that of placebo group showed a significant efficiency of 1.04%, an effective rate of 46.88%, and a total effective rate of 47.92%, suggesting statistically significant differences between the two groups (*P* < 0.001) (Figure 5; Table 4).

**TABLE 4:** The effective rate of dyspepsia syndrome.

Index (n, %)	Placebo (n = 96)	Elian Granule (n = 97)	*χ* ^2^	*P*
Clinically cured	0 (0)	0 (0)	34.253	<0.001
Significantly effective	1 (1.04)	10 (10.31)		
Effective	45 (46.88)	73 (75.26)		
Ineffective	50 (52.08)	14 (14.43)		

**FIGURE 6:**
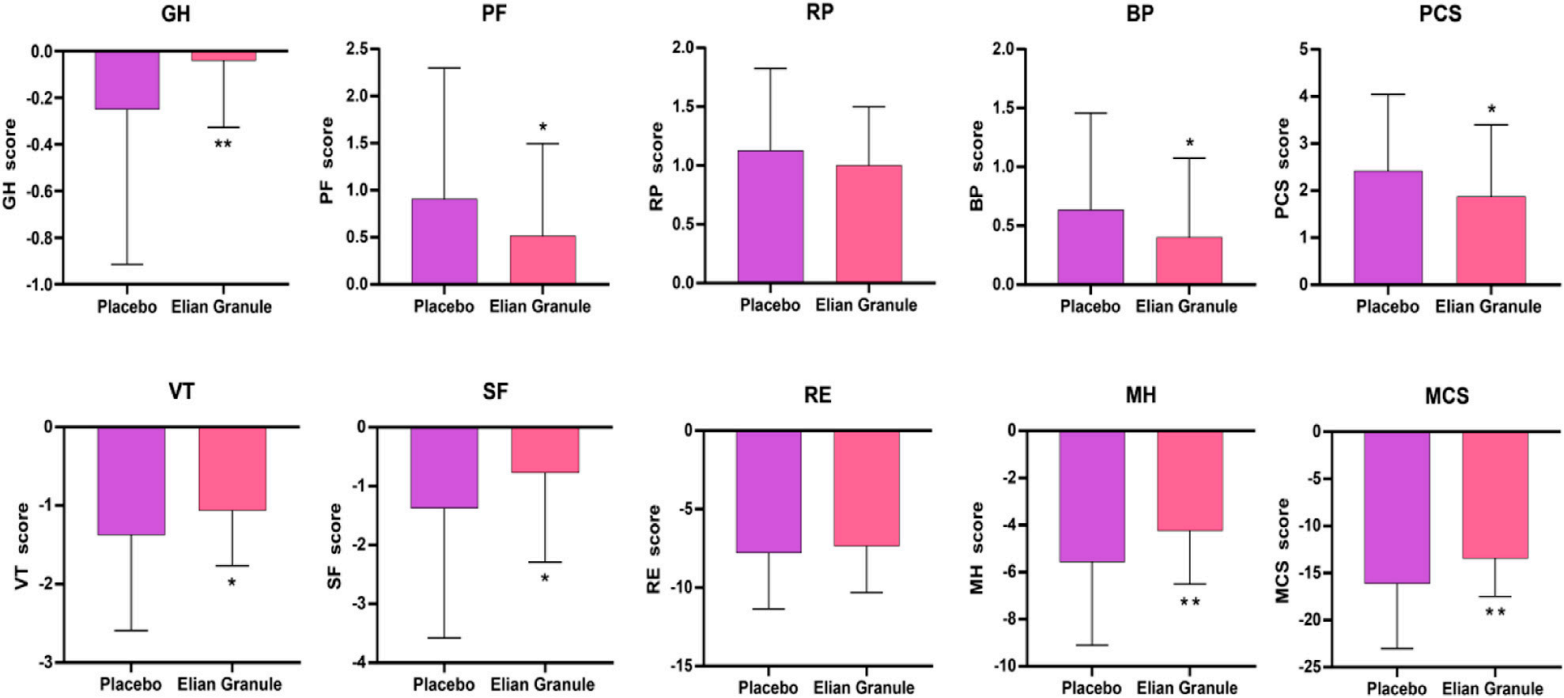
Quality of life (QOL) score at 24w, compared with the placebo group, ^*^ *P* < 0.05; ^**^ *P* < 0.01). GH, general health; PF, physical functioning; RP, role-physical; BP, bodily pain; PCS, physical component summary; VT, vitality; SF, social functioning; RE, role-emotional; MH, mental health; MCS, mental component summary.

In terms of QOL scores, after the treatment, GH, PF, BP, and PCS, and VT, SF, MH, and MCS in Elian Granule group were significantly improved compared with placebo group (*P* < 0.05, *P* < 0.01) (Figure 6; Table 5).

**TABLE 5:** Comparison of PCS and MCS scores.

Index	Placebo (n = 96)	Elian Granule (n = 97)	*P*
PCS (mean [SD])	2.42 (1.63)	1.88 (1.52)	0.018^*^
GH	−0.25 (0.66)	−0.04 (0.29)	0.005^**^
PF	0.91 (1.39)	0.52 (0.98)	0.025^*^
RP	1.13 (0.70)	1.00 (0.50)	0.155
BP	0.64 (0.82)	0.40 (0.67)	0.032^*^
MCS (mean [SD])	−16.10 (6.90)	−13.43 (4.05)	0.001^**^
VT	−1.38 (1.22)	−1.06 (0.70)	0.030^*^
SF	−1.38 (2.20)	−0.77 (1.52)	0.028^*^
RE	−7.78 (3.57)	−7.35 (2.95)	0.362
MH	−5.57 (3.53)	−4.25 (2.25)	0.002^**^

Note: Compared with the placebo group, ^*^ *P* < 0.05, ^**^ *P* < 0.01.

### 3.4 Safety analysis

Among the 239 participants in the safety analysis set (119 in the Elian Granule group, and 120 in the placebo group), the incidence of AEs was 1.68% (2/119) in the Elian Granule group and 1.67% (2/120) in the placebo group. One patient from each group withdrew due to AE (diarrhea). All reported AEs during the study were listed in Table 6. No severe AEs in were reported in either group. Additionally, no adverse effects were observed on ECG, blood routine (RBC, WBC, PLT, and HGB), urine routine (urine sugar, urine protein, urine red blood cell, and urine white blood cell), liver function (ALT, AST, ALP, γ-GT, and TBIL), or renal function (Scr, BUN, and GFR) in either group (Supplementary Material 2).

**TABLE 6:** Adverse event (AE).

Event (n, %)	Placebo (n = 120)	Elian Granule (n = 119)
Diarrhea	1 (0.83)	1 (0.84)
Insoluble drug	1 (0.83)	0 (0.00)
Rash and itch	0 (0.00)	1 (0.84)

## 4 Discussion

Traditional Chinese medicine (TCM) offers distinct advantages in treating chronic atrophic gastritis (CAG). A randomized, double-blind clinical trial of Moluodan involving 130 CAG patients with dysplasia, utilizing standardized biopsy techniques, reported effective rates of 34.6% for atrophy and 23% for intestinal metaplasia after 6 months of treatment (Tang et al., 2016). Another randomized, double-blind trial of Gaierkang tablets in 108 CAG patients demonstrated a 51.7% effective rate for atrophy/intestinal metaplasia after 3 months (Chen et al., 2023). Previous research from our group indicated that Elian Granule could effectively reduce gastric mucosal inflammation, atrophy, and enteritis (Cong et al., 2015; Gu et al., 2015). This trial expands on our prior work, offering deeper insight into Elian Granule’ therapeutic effects. Designed as a large-scale, national multi-center study with a double-blind, randomized, placebo-controlled structure, the trial reveals Elian Granule can achieve effective rates of 76.29% for atrophy and 62.89% for intestinal metaplasia in CAG patients, underscoring their significant pathological therapeutic impact. Aligned with the “Special Provisions for the Registration Management of TCM”, this study targets CAG through Elian Granule precisely addressing the core pathogenesis of spleen deficiency, dampness stagnation, blood stasis, and heat interconnection. Over three decades of clinical use, Elian Granule has proven their significant benefits and broad applicability across various syndromes.

The accuracy of pathological tissue sampling has always been a recognized difficulty and controversy. Accurate pathological diagnosis and efficacy evaluation depend on standardized biopsy sampling. Due to the localized distribution of CAG lesions, it is difficult to obtain specimens accurately at the same site using conventional biopsy techniques. This complicates the comparison and undermines the reliability of the biopsy’s accuracy before and after therapy, therefore impeding the scientific rigor and dependability of research findings. Moreover, in clinical practice, it is more common to take two to three pieces of pathology under endoscopy, which also adds some difficulties to clinical observation and pathological efficacy evaluation. The current consensus in diagnosing and grading the risk of gastric mucosal precancerous diseases is the OLGA/OLGIM staging (Banks et al., 2019; Pimentel-Nunes et al., 2019). The OLGA/OLGIM staging system, internationally recognized for standardizing gastric mucosal lesion classification, ensures consistent diagnoses, reduces variability, and enhances communication accuracy. It effectively stratifies patients by their risk of gastric cancer progression, a key advantage of our trial. To ensure accurate pathological efficacy assessment, we trained endoscopists across multiple centers to follow OLGA/OLGIM staging during sampling, minimizing subjective detection bias (Dixon et al., 1996; Varbanova et al., 2016; Isajevs et al., 2014). Furthermore, pathological evaluation is a crucial component of assessing clinical effectiveness. Rigorous diagnostic protocols, including regular random sampling and a team of experienced senior physicians reviewing pathological results using a standardized blind method, further enhanced histological evaluation precision.

Dyspepsia, a primary clinical manifestation of CAG, significantly reduces patients’ quality of life and strains healthcare resources (Rodriguez-Castro et al., 2018; Miceli et al., 2012; Li et al., 2018). TCM excels in treating CAG and improving dyspeptic symptoms. From the perspective of total dyspepsia symptom score, the 4w, 12w, and 24w follow-up results in Elian group were all significantly better than the placebo group, indicating that Elian Granule can significantly improve dyspepsia symptoms with long-term efficacy. Once the gastric mucosa is damaged, gastric acid secretion is subsequently reduced, leading to a weakened protective capacity of the gastric mucosa. This makes food unable to be completely digested and remains in the stomach, causing upper abdominal pain. Elian Granule can alleviate epigastric pain at 4w, 12w, and 24w, which may be related to promoting gastrointestinal motility, and improving gastrointestinal digestive function. The delayed digestion of food in the stomach, resulting from inadequate gastric acid secretion, also leads to prolonged retention of food and a deceleration in gastric peristalsis, eventually causing symptoms such as epigastric distension and epigastric discomfort. Atrophy reduces gas excretion from the gastric wall, and food cannot be completely decomposed during digestion, leading to the continuous production of gas in the gastrointestinal tract, resulting in uncomfortable symptoms such as belching (Li et al., 2018). In our present study, abdominal distension, epigastric discomfort, and belching were relieved after the treatment of Elian Granule at 4w and 12w. The abdominal distension and belching were also relieved at 24w. The main reason may be that the active ingredients of Curcuma phaeocaulis Valeton (Ezhu), Citrus reticulata Blanco (Chenpi), and Poria cocos (Schw.) Wolf (Fuling) reduce gastrointestinal inflammation and regulate gastrointestinal motility (Gong et al., 2023; Lin et al., 2024; Nie et al., 2020). Reduced gastric capacity as a result of gastric mucosa atrophy and muscle layer weakening is a common cause of early satiety. Delayed gastric emptying, reduced gastric acid secretion, and an irregular diet can all cause early satiety (Lahner et al., 2018). Elian Granule alleviating early satiety at 4w, 12w, and 24w might be related to the fact that Elian Granule regulates the gastrointestinal system, promotes gastrointestinal motility, and increases appetite, thereby relieving symptoms. Besides, if gastric emptying is delayed, food will remain in the stomach for a longer time, and gastric acid secretion time will also increase accordingly. Gastric acid refluxes into the esophagus, damaging the esophageal mucosa and causing symptoms such as acid reflux and heartburn. Elian Granule could improve acid reflux and heartburn for 4w and 12w, and it continued to alleviate acid reflux after 24 weeks of treatment. This may be related to the fact that the active ingredients in Curcuma phaeocaulis Valeton (Ezhu) stimulate the gastric mucosa to secrete mucus, form a mucus membrane, protect the gastric mucosa, and reduce gastric acid corrosion and irritation (Lin et al., 2024). In conclusion, Elian Granule has demonstrated significant efficacy in alleviating dyspeptic symptoms in patients with CAG. This improvement not only reduces the frequency and severity of symptoms such as epigastric pain, epigastric discomfort, and belching but also addresses the psychological burden associated with chronic illness. By effectively managing these symptoms, patients experience reduced anxiety related to their health condition, leading to enhanced emotional wellbeing. Furthermore, the alleviation of symptoms allows patients to resume normal daily activities with greater ease, improving their overall quality of life.

Ancient Chinese doctors always combined patients’ living conditions, social environment, physical functions, psychological state and other factors to regulate patients’ physical state, which is the embodiment of the “holistic concept” of TCM. In addition, the “syndrome” of “syndrome differentiation and treatment” in TCM includes not only the symptoms and signs of the disease, but also the patient’s mental health, which is consistent with the basic connotation of quality of life. It can be seen that although the concept of quality of life does not exist in Chinese medicine, the connotation of quality of life has been reflected in ancient medical books and practice. Nowadays, quality of life is a comprehensive indicator that comprehensively reflects an individual’s health status. It is a daily functional description of a patient’s physical, psychological and social responses to disease and treatment. It is considered an important aspect in evaluating the clinical value of treatments. There are many types of scales used clinically to evaluate patients’ quality of life. In this study, we used the internationally recognized SF-12 for measurement, which included eight dimensions and was mainly divided into two categories: physical health level and mental health level (Lam et al., 2013; Tucker et al., 2014). A cross-sectional study of atrophic gastritis showed that the atrophic gastritis group was significantly more impaired in physiological functions than the functional gastrointestinal disease group, and poorer physical health scores might be related to the presence of severe frailty (Miceli et al., 2021). It can be seen that as age increases, the function of the spleen (TCM meaning) and stomach gradually weakens, and the physical functions decrease accordingly. The scores of GH, PF, BP, and PCS in the Elian Granule group were significantly improved after treatment, suggesting that Elian Granule could alleviate the physical symptoms of CAG patients. This was considered to be related to the fact that SiJunZi decoction in the prescription could tonify the spleen, enriched qi, and strengthened the body. In today’s society, the rapid pace of life, and emotional distress have become important factors affecting the occurrence, development, and evolution of diseases. In particular, CAG patients often suffer from anxiety or depression due to fear of cancer. A cross-sectional study of atrophic gastritis found that compared with the control group, the atrophic gastritis group had significantly higher impairments in all subscales of quality-of-life evaluation (Miceli et al., 2021). The gastrointestinal tract possesses a substantial population of neuronal cells, ranking only second to the central nervous system in terms of abundance, and has dual endocrine and neurological functions. Therefore, there is a saying that the gastrointestinal tract is the second largest emotional organ. At the same time, inflammatory mediators stimulate the cerebral cortex, thereby affecting human emotions, often manifested as anxiety, depression, etc. (Agirman et al., 2021). Zhao et al. have argued that the proportion of CAG patients with psychological disorders was as high as 54.5%, which significantly affected the patient’s quality of life and prognosis, and the anxiety and depression of CAG patients were closely related to their symptoms and the severity of gastric mucosal precancerous lesions (Zhao et al., 2018). The scores of VT, SF, MH and MCS in the Elian Granule group were significantly improved after treatment, suggesting that Elian Granule could improve the psychological state of patients, which was considered to be related to the fact that Elian Granule relieves patients’ gastrointestinal symptoms and thereby achieves benign brain-gut interaction. Thus, Elian Granule enhances quality of life by alleviating physical symptoms and reducing anxiety, emphasizing the importance of integrating physical and mental treatment in clinical CAG management. As the ancient medical book “*Yi Zong Jin Jian*” advises, “In treating stomach illnesses, … calm the mind, eliminate distractions, and patiently await the return of vital energy to normal”.

Placebo-controlled randomized studies are the gold standard for evaluating the intervention efficacy in clinical trials (Xie et al., 2012). Placebo aim to minimize psychological and other factors among relevant participants such as researchers, subjects and evaluators to the greatest extent, confirming experimental drugs’ efficacy and safety (Colloca and Barsky, 2020). In this study, the placebo showed objective effects, with effective rates of 48.96% for atrophy, 34.38% for intestinal metaplasia, and 47.92% for dyspepsia symptoms, respectively. Regarding the placebo effect: (1) A very low-dose experimental drug in the placebo is no longer a classic placebo control, but from the perspective of clinical trials, the experimental drug and placebo are in different dosage groups, and can still be utilized to verify the effectiveness of the experimental drug under the premise of complying with the superiority trial design. Therefore, in this study, 5% of the original Elian Granule was added to make a placebo, complying with clinical trials requirements. (2) Long-term low-dose placebo intervention may produce additive effects, where multiple functional drugs’ doses can be superimposed for synergistic enhancement, which is called “superimposed dosages”. Although each single medicine’s dose is small, cumulative effects arise from dose superposition and long-term use. The placebo, containing 5% of the original drug, may exert pharmacological effects over the 24-week CAG treatment. (3) Placebo effect research indicates it comprises physiological effects related to drug taste, non-specific effects linked to spontaneous recovery, and true placebo effects tied to belief in the drug’s efficacy (Eccles, 2002). A large amount of evidence shows the placebo effect is a real psychobiological phenomenon with applications in various conditions such as depression, chronic pain, anxiety, cough, IBS, Parkinson’s disease, and epilepsy (Price et al., 2008; Zubieta and Stohler, 2009; Petrovic et al., 2002; Zhang et al., 2011). In conclusion, the experimental group’s effect can be independent of the placebo effect, but the placebo effect depends on the experimental group.

## 5 Conclusion

**FIGURE 7:**
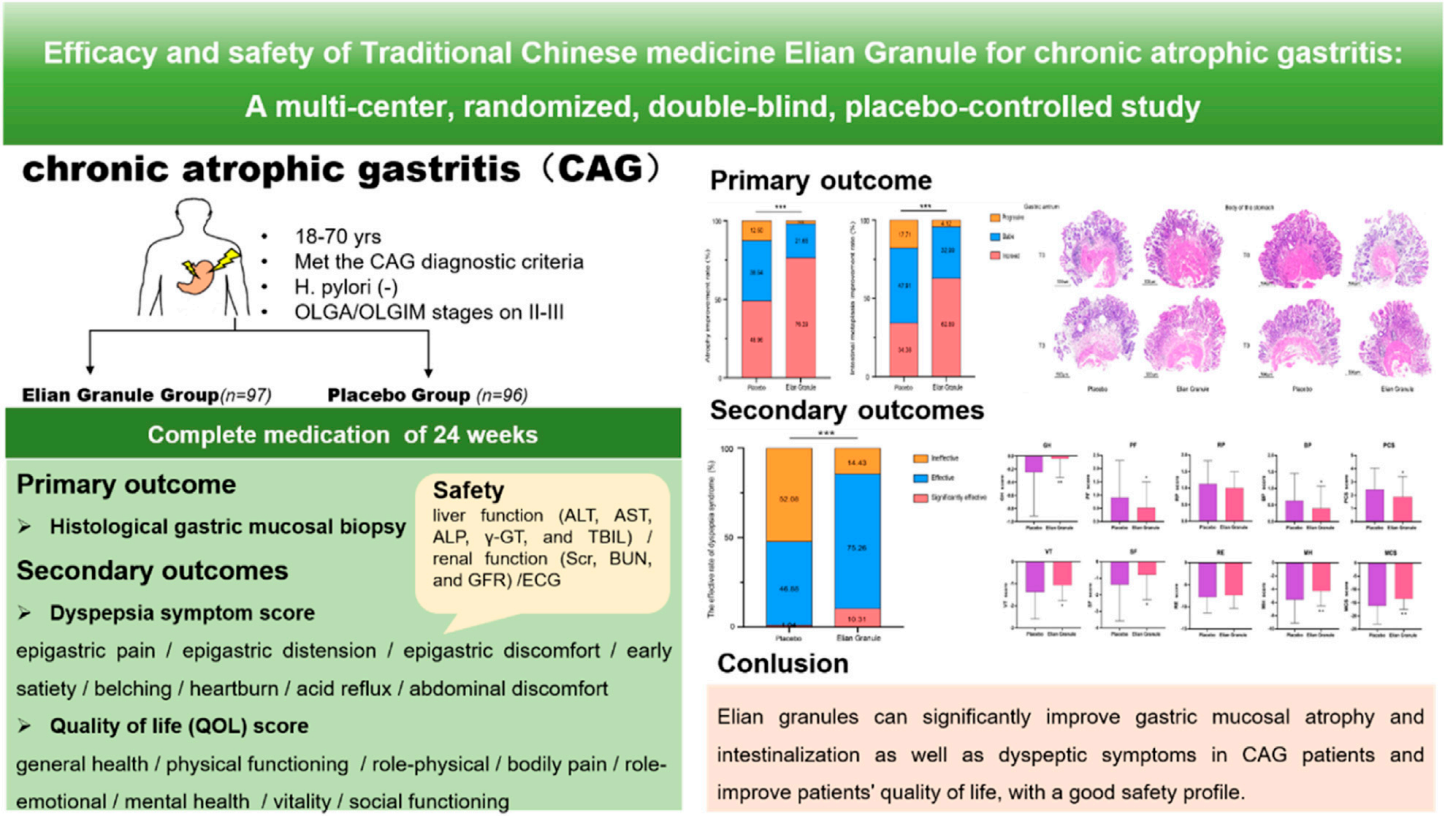
Elian Granule can significantly improve gastric mucosal atrophy and intestinalization as well as dyspeptic symptoms in CAG patients and improve patients’ quality of life, with a good safety profile.

This study, for the first time, has carried out a national, multicenter, double-blind, randomized, placebo-controlled clinical trial to evaluate the therapeutic effects and safety of Elian Granule on CAG. The results indicate that Elian Granule can significantly improve gastric mucosal atrophy and intestinal metaplasia in CAG patients, relieve dyspeptic symptoms, enhance patients’ quality of life, and demonstrate good safety. This trial not only confirms the clinical value of Elian Granule in treating CAG but also offers high-quality evidence-based medical support for TCM in CAG therapy (Figure 7).

## Data Availability

The original contributions presented in the study are included in the article/Supplementary Material, further inquiries can be directed to the corresponding authors.
