# Supplementary material for: Efficacy and safety of traditional Chinese medicine Elian Granule for chronic atrophic gastritis: a multi-center, randomized, double-blind, placebo-controlled study
Source: Front Pharmacol. 2025 Apr 28;16:1545313. doi: 10.3389/fphar.2025.1545313 (PMC12066464; doi:10.3389/fphar.2025.1545313)
Supplement: Supplementary file 1 [file DataSheet2.pdf]

Item	Index	Elian Granule				Placebo				χ^2	*P*
		Normal	Abnormal but no significant	Abnormal with significant	No tested	Normal	Abnormal but no significant	Abnormal with significant	No tested		
**Blood cell count**	RBC(10/L)										
	T0	114	5	0	0	110	8	0	2	2.760	0.252
	T3	93	4	0	0	94	2	0	0	0.667	0.683
	WBC(10/L)										
	T0	110	6	3	0	112	6	0	2	5.014	0.171
	T3	89	8	0	0	90	6	0	0	0.286	0.783
	PLT(10/L)										
	T0	114	5	0	0	109	9	0	2	3.251	0.197
	T3	91	6	0	0	88	8	0	0	0.331	0.592
	HGB(g/L)										
	T0	114	4	0	1	108	10	0	2	3.063	0.216
	T3	93	4	0	0	91	5	0	0	0.128	0.747
**Urinalysis**	Urine glucose(mmol/L)										
	T0	113	2	0	4	115	3	0	2	0.880	0.644
	T3	95	1	1	0	94	1	1	0	0.000	1.000
	Urine protein(g/L)										
	T0	105	10	0	4	106	12	0	2	0.849	0.654
	T3	88	9	0	0	84	12	0	0	0.516	0.498
	Urine erythrocyte(N/HP)										
	T0	102	11	2	4	102	16	0	2	3.588	0.309
	T3	85	11	1	0	82	13	0	1	2.215	0.529
	Urinary leukocytes(N/HP)										
	T0	106	8	1	4	109	6	3	2	1.990	0.574
	T3	92	5	0	0	91	4	0	1	1.111	0.574
**Liver and renal function**	ALT(U/L)										
	T0	112	7	0	0	112	7	0	1	0.996	0.608
	T3	91	6	0	0	89	6	1	0	1.017	0.601
	AST(U/L)										
	T0	110	9	0	0	113	6	0	1	1.636	0.441
	T3	92	5	0	0	91	5	0	0	0.000	1.000
	ALP(U/L)										
	T0	110	8	0	1	116	3	0	1	2.428	0.297
	T3	89	8	0	0	94	2	0	0	3.372	0.100
	γ-GT(U/L)										
	T0	109	10	0	0	113	6	0	1	2.068	0.356
	T3	89	5	0	3	90	4	0	2	0.312	0.856
	TBIL(μmol/L)										
	T0	109	10	0	0	113	4	0	3	5.639	0.060
	T3	90	6	0	1	83	10	1	2	2.611	0.455
	Scr(μmol/L)										
	T0	113	6	0	0	110	8	1	1	2.322	0.508
	T3	93	4	0	0	92	4	0	0	0.000	1.000
	BUN(mmol/L)										
	T0	117	2	0	0	116	3	0	1	1.200	0.549
	T3	97	0	0	0	93	3	0	0	3.079	0.121
	GFR(ml/min)										
	T0	109	10	0	0	101	18	0	1	3.586	0.166
	T3	88	9	0	0	80	16	0	0	2.336	0.139
**ECG**	T0	74	42	1	2	74	44	2	0	2.376	0.498
	T3	52	44	0	1	55	38	2	1	2.518	0.472
