# Supplementary material for: Efficacy and safety of traditional Chinese medicine Elian Granule for chronic atrophic gastritis: a multi-center, randomized, double-blind, placebo-controlled study
Source: Front Pharmacol. 2025 Apr 28;16:1545313. doi: 10.3389/fphar.2025.1545313 (PMC12066464; doi:10.3389/fphar.2025.1545313)
Supplement: Supplementary file 2 [file DataSheet3.pdf]

上海中医药大学附属曙光医院伦理委员会
IRB of Shuguang Hospital affiliated with Shanghai University of TCM

伦理审查批件
Approval Notice

批件号 Approval Number: 2020-834-41-01

项目名称<br>Protocol Title	菝连颗粒治疗慢性萎缩性胃炎的随机、双盲、安慰剂对照、多中心临床试验
申办者 Sponsor	国家中医药管理局中医药循证能力建设项目
合同研究组织 CRO	NA
CFDA 批件号 Approval No.by CFDA	NA
主要研究者 Principle Investigator	脾胃病科 凌江红
审查方式/审查日期<br>Type of Review/Date	■ 会议审查 Meeting Review / 2020 年 6 月 24 日<br>■ 快速审查 Expedited Review / 2020 年 7 月 13 日
会议地点 MeetingLocation	行政二楼 C2007 景观房
会议出席情况 Meeting Attendance	应到 Total 11 人，出席 Attendance 7 人，回避 Avoidance 0 人

已批准的文件（含版本号） Documents approved with Version No.:

1. 初始审查申请表
2. 其他中心主要研究者名单
3. 项目任务书
4. 研究方案（版本号：1.2，版本日期：2020 年 6 月 27 日）
5. 知情同意书（版本号：1.2，版本日期：2020 年 6 月 27 日）
6. 招募广告（版本号：1.2，版本日期：2020 年 6 月 27 日）
7. 病例报告表（版本号：1.2，版本日期：2020 年 6 月 27 日）
8. 主要研究者履历与伦理相关培训证书

审查决定 Decision for this proposal	■ 同意 Approved		
跟踪审查频率 Continuing review frequency	□ 3 个月	□ 6 个月	■ 12 个月
批件有效期 the Approval Valid	2020 年 7 月 13 日~2021 年 7 月 12 日		

委员会主席签字 Signature:

批准日期 Date: 2020 年 7 月 13 日

（伦理委员会盖章 Seal）

上海中医药大学附属曙光医院伦理委员会

扫描全能王 创建

莪连颗粒 1.3 修改明细

说明：本次修改为在项目启动会后与分中心进一步讨论后所做修改。

方案：

1. 合作单位：**去除**“广西中医药大学附属瑞康医院、山东郯城县第一人民医院”，**增加**“长春中医药大学附属医院、安徽中医药大学二附院、浦东光明中医医院、安徽医科大学附属第二医院、六安市中医院、麻城市人民医院”。
2. 版本号**改**1.3，制定日期**改**9月23日。
3. 研究任务与方案：
   a) “1.1样本量估算”**补充**“竞争性入组，各中心承担不少于16例。”并在“附件1：各中心病例分配表”中修改相关药物编号。
   b) “3.1试验用药物”、“3.3治疗方案”**补充**药物具体规格“12.1g/袋”；删除单位“剂”，统一为“袋”（一剂4袋）。
   c) “3.2.6”、“3.6.1”、“8.数据管理”、“10.2临床试验记录”、“10.8监查员及临床试验监察”、“13.资料保存与总结”中**修改**CRF病例报告表相关内容为EDC，本试验已经建立EDC，入组病例收集方式取消纸质版CRF，全部采用eCRF收集。并单独列出附件5、6、7，以供分中心打印使用，保留原始材料。
   d) “4.2基线（0天）”**删除**“（1）采集基本病史：再次审核入选/排除标准。（2）有效性指标：记录临床症状，进行症状积分；生活质量评分。”（筛选期已完成上述内容收集，取消了基线的重复收集）
4. **删除**“附件4：药物性肝病处理参考预案（SOP）”、“附件5：药物性肾损害处理参考预案（SOP）”、“附件6：心血管不良事件处理参考预案（SOP）”、“附件7：SAE报告相关单位联系方式”、“附件8：严重不良事件报告表（SAE）”。**增加**“附件：消化不良症状积分表”、“附件：生活质量评分表”、“附件：中医证候分型表”。并据上述方案修改内容及CRF表相关内容**调整**“附件：试验流程图”。
5. 方案内容未变，**文字上的表述及细节错误修订**：
   a) “4.访视计划及试验流程”中“4.1筛选期”**增加**“中医证候分型”，尿妊娠试验后**增加**说明“（适用者）”；“4.3”、“4.4”**修改**文字表述为“检查患者记录卡记录情况并评估，清点药物并发放试验用药物”；“4.5”**删除**“大便常规+潜血”（1.2版本已经删除，此处为文字漏删）、**增加**“回收并检查患者记录卡并临床评估；试验结束总结。”
   b) “5.1.1主要疗效指标”**修改**“异型增生”表述为“上皮内瘤变”。

CRF：

1、细节标点符号**修改**。

2、版本号**改**1.3，制定日期**改**9月23日。

3、根据方案**增加**分中心。

4、进一步**明确**了一般资料、体格检查、合并用药情况、入排标准、消化不良症状积分、中医证候分型、呼气试验、药品的回收/清点、检查患者记录卡并临床评估等项目的访视时

间。(主要在筛选期已经采集过的信息，取消了基线的重复采集)。

5、 内容未变，**文字上的表述及细节错误修订**：

   a) “试验流程图”中**删去**第4、5、6次访视（方案1.2中以删除，此处为漏删)。

   b) 入排标准与方案保持一致（与1.2方案一致，未做修改)。

6、 筛选期**增加**呼气试验检测回报。

7、 根据方案中每次访视内容的**修改**进行CRF表的修改。

上海中医药大学附属曙光医院伦理委员会

IRB of Shuguang Hospital affiliated with Shanghai University of TCM

关于“我连颗粒治疗慢性萎缩性胃炎的随机、双盲、安慰剂对照、多中心临床试验”伦理审查意见

Notification of IRB Meeting Minutes

项目编号：2020-834-41

项目名称：我连颗粒治疗慢性萎缩性胃炎的随机、双盲、安慰剂对照、多中心临床试验

项目来源：国家中医药管理局中医药循证能力建设项目

研究负责单位： 上海中医药大学附属曙光医院

主要研究者： 脾胃病科 凌江红

研究参加单位：上海中医药大学附属岳阳医院、杭州市中医院、山西中医药大学附属医院、广西中医药大学第一附属医院、广西中医药大学附属瑞康医院、江苏省泰州市第二人民医院、山西省晋城大医院、山东郯城县第一人民医院、青海红十字医院

审查日期：2020-11-4　　审查地点：曙光东院

审查类型：修正案审查　　审查方式：快速审查

审查委员：朱梅萍

审查文件：

1. 修正方案伦理审查申请表
2. 研究方案（版本号：1.3，版本日期：2020 年 9 月 23 日）
3. 病例报告表（版本号：1.3，版本日期：2020 年 9 月 23 日）

审查意见：

根据我国国家食品药品监督管理局《药物临床试验伦理审查工作指导原则》(2010)、《药物临床试验质量管理规范》(2003)、世界医学会《赫尔辛基宣言》、以及国际医学科学组织委员会《人体生物医学研究国际道德指南》等的伦理原则，经本伦理委员会审查决定：

同意。

请对研究方案、知情同意书等相应文件作上述修改/补充，并将修改/补充后的文件提交伦理委员会审查批准后执行。如对审查意见有不同观点，请书面向伦理委员会主任委员反映。

委员会主席签字：

日期：2020-11-4

上海中医药大学附属曙光医院伦理委员会（盖章）

扫描全能王 创建

莪连颗粒治疗慢性萎缩性胃炎的随机、双盲、安慰剂对照、多中心临床试验

V1.4 修改明细

方案修改明细：

1. 合作单位：**去除**“泰州市第二人民医院、晋城大医院”，**增加**“上海市光华中西医结合医院、上海市嘉定区中医医院”，并在其他涉及处进行变更。
2. 版本号**改V1.4；** 制定日期**改2021年10月19日**。
3. 研究任务与方案：

   7.8.2 处理程序“药物性肝病处理参考预案、药物性肾损害处理参考预案、请见附件6、附件7。心血管不良事件评定标准，请见附件8”**修改为** “药物性肝病处理参考预案、药物性肾损害处理参考预案，请见附件8、附件9。心血管不良事件评定标准，请见附件10”
4. **新增**“附件8：药物性肝病处理参考预案（SOP）”、“附件9：药物性肾损害处理参考预案（SOP）”、“附件10：心血管不良事件处理参考预案（SOP）”。
5. **附件1 “**(09)泰州市第二人民医院**”**修改为“上海市光华中西医结合医院”；“（11）晋城大医院”修改为“上海市嘉定区中医医院”。
6. 方案内容未变，**文字上的表述及细节错误修订**：
   a) **方案摘要** “病理属于OLGA分期Ⅱ、Ⅲ 期”**修改为**“病理属于OLGA/OLGIM分期Ⅱ、Ⅲ 期”；“黏膜定标活检组织学检查”**修改为**“黏膜活检组织学检查”
   b) 2.3 纳入标准 “病理属于 OLGA 分期Ⅱ、Ⅲ 期”**修改为**“病理属于 OLGA/OLGIM 分期Ⅱ、Ⅲ 期”
   c) **附件2 2.胃黏膜组织学检查**“并行黏膜定标活检”**去掉**“定标”两字。
   d) **格式调整：全文两端对齐**

CRF表修订说明：

1、版本号**改**1.4，制定日期**改**2021年10月19日。

2、根据方案**增减**分中心。

上海中医药大学附属曙光医院伦理委员会

IRB of Shuguang Hospital affiliated with Shanghai University of TCM

关于"莪连颗粒治疗慢性萎缩性胃炎的随机、双盲、安慰剂对照、多中心临床试验"伦理审查意见

Notification of IRB Meeting Minutes

项目编号：2020-834-41

项目名称：莪连颗粒治疗慢性萎缩性胃炎的随机、双盲、安慰剂对照、多中心临床试验

项目来源：国家中医药管理局中医药循证能力建设项目

研究负责单位： 上海中医药大学附属曙光医院

主要研究者： 脾胃病科 凌江红

研究参加单位：上海中医药大学附属岳阳医院、杭州市中医院、山西中医药大学附属医院、广西中医药大学第一附属医院、广西中医药大学附属瑞康医院、江苏省泰州市第二人民医院、山西省晋城大医院、山东郯城县第一人民医院、青海红十字医院

审查日期：2021.10.26 审查地点：曙光东院

审查类型：修正案审查 审查方式：快速审查

审查委员：朱梅萍

审查文件：

1. 修正方案伦理审查申请表
2. 研究方案（版本号：1.4，版本日期：2021 年 10 月 19 日）
3. 病例报告表（版本号：1.4，版本日期：2021 年 10 月 19 日）

审查意见：

根据我国国家食品药品监督管理局《药物临床试验伦理审查工作指导原则》、《药物临床试验质量管理规范》、世界医学会《赫尔辛基宣言》、以及国际医学科学组织委员会《人体生物医学研究国际道德指南》等的伦理原则，经本伦理委员会审查决定：

同意。

请对研究方案、知情同意书等相应文件作上述修改/补充，并将修改/补充后的文件提交伦理委员会审查批准后执行。如对审查意见有不同观点，请书面向伦理委员会主任委员反映。

委员会主席签字：[signature]

日期：2021.10.26

上海中医药大学附属曙光医院伦理委员会（盖章）

上海中医药大学附属曙光医院 伦理委员会

扫描全能王 创建
