# Supplementary material for: Efficacy and safety of traditional Chinese medicine Elian Granule for chronic atrophic gastritis: a multi-center, randomized, double-blind, placebo-controlled study
Source: Front Pharmacol. 2025 Apr 28;16:1545313. doi: 10.3389/fphar.2025.1545313 (PMC12066464; doi:10.3389/fphar.2025.1545313)
Supplement: Supplementary file 3 [file DataSheet1.pdf]

Outcome	Placebo (n=96)	Elian granule (n=97)	*P*	Group Effect *F*	*P*	Time Effect *F*	*P*	Group × Time Effect *F*	*P*
Total Dyspepsia symptom score (mean [SD])				7.20	0.008	153.63	<0.001	14.58	<0.001
T0	17.34 (5.75)	18.57 (5.90)	0.146						
T1	15.50 (4.89)	12.72 (4.38)	<0.001						
T2	13.65 (4.52)	11.43 (3.20)	<0.001						
T3	12.15 (4.00)	10.44 (2.40)	<0.001						
Epigastric pain score (mean [SD])				6.62	0.011	80.15	<0.001	11.58	<0.001
T0	2.27 (1.13)	2.46 (1.16)	0.244						
T1	2.10 (1.07)	1.61 (0.74)	<0.001						
T2	1.83 (0.95)	1.40 (0.62)	<0.001						
T3	1.57 (0.84)	1.25 (0.48)	0.001						
Epigastric distension score (mean [SD])				3.11	0.079	68.35	<0.001	8.12	<0.001
T0	2.29 (1.12)	2.53 (1.14)	0.152						
T1	2.00 (0.93)	1.68 (0.78)	0.011						
T2	1.80 (0.91)	1.54 (0.71)	0.025						
T3	1.67 (0.83)	1.33 (0.51)	<0.001						
Epigastric discomfort score (mean [SD])				5.28	0.023	80.42	<0.001	5.57	0.002
T0	2.57 (1.09)	2.63 (1.18)	0.732						
T1	2.33 (1.02)	1.88 (0.94)	0.001						
T2	2.03 (0.90)	1.66 (0.73)	0.002						
T3	1.70 (0.88)	1.49 (0.61)	0.065						
Early satiety score (mean [SD])				1.45	0.230	44.77	<0.001	6.03	0.002
T0	1.80 (1.10)	2.05 (1.25)	0.144						
T1	1.66 (1.01)	1.38 (0.81)	0.039						
T2	1.43 (0.79)	1.18 (0.41)	0.006						
T3	1.24 (0.58)	1.10 (0.31)	0.041						
Belching score (mean [SD])				4.21	0.042	56.44	<0.001	4.79	0.007
T0	2.23 (1.22)	2.34 (1.16)	0.518						
T1	1.93 (0.92)	1.57 (0.75)	0.003						
T2	1.80 (0.87)	1.47 (0.69)	0.004						
T3	1.52 (0.74)	1.31 (0.58)	0.029						
Heartburn score (mean [SD])				2.08	0.151	37.06	<0.001	4.91	0.005
T0	2.01 (1.05)	2.18 (1.16)	0.303						
T1	1.79 (0.94)	1.48 (0.75)	0.013						
T2	1.64 (0.77)	1.41 (0.73)	0.040						
T3	1.55 (0.77)	1.37 (0.62)	0.072						
Acid reflux score (mean [SD])				3.58	0.060	38.38	<0.001	4.83	0.004
T0	2.01 (1.04)	2.13 (1.13)	0.431						
T1	1.94 (0.90)	1.57 (0.80)	0.003						
T2	1.60 (0.76)	1.39 (0.67)	0.041						
T3	1.53 (0.78)	1.30 (0.63)	0.024						
Abdominal discomfort score (mean [SD])				0.57	0.450	67.02	<0.001	1.67	0.187
T0	2.16 (1.25)	2.25 (1.26)	0.614						
T1	1.75 (0.94)	1.56 (0.89)	0.144						
T2	1.51 (0.81)	1.38 (0.60)	0.210						
T3	1.38 (0.77)	1.29 (0.63)	0.395						
